# Editorial: Interstitial Lung Disease Around the World

**DOI:** 10.3389/fmed.2022.865334

**Published:** 2022-03-18

**Authors:** Marlies Wijsenbeek, Joyce S. Lee, Zarir Udwadia, Michael Kreuter

**Affiliations:** ^1^Centre of Excellence for Interstitial Lung Diseases and Sarcoidosis, Department of Respiratory Medicine, Erasmus Medical Centre (MC), University Hospital Rotterdam, Rotterdam, Netherlands; ^2^Department of Medicine, University of Colorado School of Medicine, Aurora, CO, United States; ^3^Hinduja Hospital and Research Center, Mumbai, India; ^4^Center for Interstitial and Rare Lung Diseases & Interdisciplinary Sarcoidosis Expert Center, Pneumology, Thoraxklinik, University of Heidelberg, Heidelberg, Germany; ^5^German Center for Lung Research (TLRC), Heidelberg, Germany

**Keywords:** Interstitial Lung Disease (ILD), idiopathic pulmonary fibrosis, pulmonary fibrosis, inequality in access to healthcare, hypersensitivity pneumonitis (HP), familial pulmonary fibrosis (FPF), air pollution, occupational and environmental exposure

Interstitial Lung Disease (ILD) comprise a broad spectrum of diseases, with different underlying pathophysiology, disease behavior, and outcomes. For the most prevalent forms of ILD such as idiopathic pulmonary fibrosis (IPF), sarcoidosis and hypersensitivity pneumonitis, international guidelines have been developed and are updated regularly to promote uniformity of diagnosis and treatment, and to enable collaborative research efforts (1–4). However, even for these well-defined diseases, heterogeneity exists in prevalence, disease behavior and outcomes around the world [hrefhttps://doi.org/10.3389/fmed.2021.751181Kaul et al.; (5–8)]. There are still many unknowns about the impact of geography on these diseases. As many ILDs are thought to originate from an external—often perpetuating—stimulus in a susceptible host, it is likely that environmental and genetic factors explain some of these differences (9). Furthermore, access to diagnosis, care and treatment options also impact disease recognition, outcome, and health related quality of life, which contributes to some notable differences throughout the world (10–14).

Although great advances have been made in recent years on understanding the pathogenesis and advancing the treatment of fibrotic ILDs and ultra-rare ILDs, only a limited part of the world has yet benefitted from these advances. Furthermore, many clinical trials as well as translational studies suffer from a lack of diversity in their study population, with a predominance of Caucasian males from the northern hemisphere included in most studies (13). Fortunately, there have been increasing efforts to expand this narrow scope. First, pharmaceutical companies are now including a broader scope of countries in their clinical trials. For example, when looking at IPF, the CAPACITY trials ran in 13 countries in Europe, Australia and North America, the INPULSIS trials in 24 countries in the Americas, Europe, Asia and Australia, and the ISABELA studies in 26 countries and covering all continents (15–17). Second, academic societies are promoting international collaborations (18). Finally, digital collaborations and communication, accelerated in part by the pandemic, have stimulated surveying and meeting people around the world about their practices and perspectives in different fields of ILD [hrefhttps://doi.org/10.3389/fmed.2021.699644Polke et al.; (19)]. We hope that these developments will result in more insights about potential pathogenetic differences and disease behavior in ILDs around the world, in addition to promoting access to diagnosis and treatment, especially for middle- and low-income countries.

Whilst prevention of ILD and reducing exposure to potential causative agents is crucial, this is a complex field. On one hand, country and even location specific interventions are necessary, whilst on the other hand, cross border agreements are crucial to reduce the important factor of air pollution (10, 20, 21). The pulmonary field should keep raising its united voice on the impact of air pollution on lung health. Meanwhile, international collaborative efforts with simple interventions could potentially have great impact, such as initiatives aimed at reducing household air pollution by supplying improved cookstoves (22).

Even though we all acknowledge the need for a more inclusive research field and more equality in access to care and treatment around the world, this is easier said than done. Barriers for conducting clinical trials include lack of financial and human capacity, ethical and regulatory obstacles, operational barriers and competing demands (23). In this issue, we aimed to achieve greater insights into the difference and similarities in ILD around the world by encouraging people from all over the world to submit their research in ILD. Furthermore, we wished to promote cross border collaborations between scientists and clinicians. This has resulted in 11 manuscripts on varying topics in ILD, written by 103 authors from 33 countries.

The manuscript of hrefhttps://doi.org/10.3389/fmed.2021.751181Kaul et al. describes the heterogeneity of ILD around the world by comparing 17 different epidemiological studies from all over the world. They demonstrate that hypersensitivity pneumonitis is more prevalent in Asia, and particularly in India, whilst in North America and Europe, IPF and sarcoidosis are more prevalent. They discuss the potential reasons and unknowns underlying such differences and call for organization of the ILD research community to develop a shared ontology for disease and collaborative epidemiological studies. That such projects are needed, but will be challenging, is further illustrated by four different studies presented in this ILD around the world issue.

These four studies illustrate that access to diagnosis and treatment, as well as disease phenotype may well-depend on the place you live. The work of van de Sar in collaboration with the European Pulmonary Fibrosis and related disorders federation, showed large differences in diagnostic access that exist between European countries (hrefhttps://doi.org/10.3389/fmed.2021.711194van der Sar et al.). Data from the well-known EMPIRE registry, comprising 10 central and eastern European countries, show clear differences in access to therapy as well as patient characteristics in a group of nearly 2,500 patients with IPF (hrefhttps://doi.org/10.3389/fmed.2021.729203Kolonics-Farkas et al.). hrefhttps://doi.org/10.3389/fmed.2021.699644Polke et al. surveyed 509 pulmonologists from 66 countries on prevention, diagnosis and treatment strategies of acute exacerbations of IPF, which yielded insights into similarities and differences across the world. Their effort also highlights the potential for large international surveys to guide future study design. Last, hrefhttps://doi.org/10.3389/fmed.2021.679487Gonzalez-Garcia et al. in four Latin American countries demonstrated the potential effect of living at high altitude on co-morbidities for patients with IPF, which supports the need for tailored diagnostic protocols depending on geography.

Health related quality of life (HRQOL) is an important outcome in the care of patients with ILD and is particularly influenced by an individual's cultural and spiritual background and values. Aronson and Suzuki provide an important and comprehensive overview of the global influences on HRQOL assessment and the different tools that exist to assess HRQOL around the world (hrefhttps://doi.org/10.3389/fmed.2021.745908Aronson and Suzuki). They also identify gaps and provide recommendations for improving HRQOL assessment across the world in ILD.

The research in this issue provides some, even if small, steps forward in our knowledge of ILD around the world. Moreover, we hope that this issue has accelerated new contacts and collaborations throughout the world. We are convinced that expanding our cross-continental networks will lead to more inclusive and conclusive research, which in the end will lead to better care for patients with ILD around the world.

## Author Contributions

All authors listed have made a substantial, direct, and intellectual contribution to the work and approved it for publication.

## Conflict of Interest

The authors declare that the research was conducted in the absence of any commercial or financial relationships that could be construed as a potential conflict of interest.

## Publisher's Note

All claims expressed in this article are solely those of the authors and do not necessarily represent those of their affiliated organizations, or those of the publisher, the editors and the reviewers. Any product that may be evaluated in this article, or claim that may be made by its manufacturer, is not guaranteed or endorsed by the publisher.
